# Treatment of Indolent Buboes

**Published:** 1880-04

**Authors:** Edmund J. Doering

**Affiliations:** Surgeon U. S. Marine Hospital Service


### ARTICLE VI.

*Treatment of Indolent Buboes.*

Every surgeon engaged in hospital practice has had his patience tried by the obstinacy of that class of buboes termed indolent or chronic. Such buboes will neither suppurate nor recede, in spite of treatment, and it is not unusual to see patients thus affected remain in hospital for six months and longer. Many surgeons, after exhausting all methods of treatment calculated either to cause the suppuration of the gland or the absorption of the inflammatory products, such as the local application of iodine, nitrate of silver, blisters or continuous pressure by a bag of shot suspended from the ceiling, have recourse finally to the knife and extirpate the gland. This latter practice unquestionably removes the trouble, but I am led to believe from a somewhat extensive experience, is very apt to be followed by a severe grade of erysipelas. Even if the patient is fortunate enough to escape an attack of erysipelas, the wound left after the removal of the gland granulates very slowly, and generally eight weeks elapse before cicatrization is completed.

While stationed in San Francisco, I treated a number of indolent buboes by introducing needles attached to a galvanic battery, as recommended by Beard and Rockwell, but with very limited success. The pain accompanying this procedure is intense, even more so than that caused by destroying the bubo with chloride of zinc pastes or other powerful caustics. During the past year I have treated all such buboes occurring in my wards at Philadelphia and my present station, Portland, Maine, by a simple method, which has afforded better results than any other treatment mentioned. Through the indurated gland I introduce from three to four setons, the materials being either a stout piece of tape or ilver wire, tied in a short loop, drawn backward and forward hrough the gland twice daily and covered with a flax-seed poulice. The setons are allowed to remain from seven to ten days, or longer, suppuration occurring generally the third day, with a rapid diminution in the size of the bubo. The results of this treatment have so far been very successful, fully 80 per cent. of the cases thus treated being cured within six weeks from the date of admission. With the exception of introducing the setons, no pain is experienced, and the patient is directed to walk about as much as he pleases, to encourage suppuration.

I am unable to state whether this treatment has been used by others, but from the results thus far attained, I am free to recommend surgeons to give this method a fair trial, believing it to be a safe and satisfactory treatment of indolent buboes.

Edmund J. Doering,
Surgeon U. S. Marine Hospital Service.

